# Acute kidney injury triggers hypoxemia by lung intravascular neutrophil retention that reduces capillary blood flow

**DOI:** 10.1172/JCI186705

**Published:** 2025-03-06

**Authors:** Yohei Komaru, Liang Ning, Carine Lama, Anusha Suresh, Eirini Kefaloyianni, Mark J. Miller, Shinichi Kawana, Hailey M. Shepherd, Wenjun Li, Daniel Kreisel, Andreas Herrlich

**Affiliations:** 1 Division of Nephrology, Department of Medicine,; 2 Division of Rheumatology, Department of Medicine,; 3 Division of Infectious Diseases, Department of Medicine,; 4 Department of Surgery, and; 5 Department of Pathology and Immunology, Washington University School of Medicine, St. Louis, Missouri, USA.; 6 VA Saint Louis Health Care System, John Cochran Division, St. Louis, Missouri, USA.

**Keywords:** Inflammation, Nephrology, Pulmonology, Monocytes, Neutrophils

## Abstract

Sterile acute kidney injury (AKI) is common in the clinic and frequently associated with unexplained hypoxemia that does not improve with dialysis. AKI induces remote lung inflammation with neutrophil recruitment in mice and humans, but which cellular cues establish neutrophilic inflammation and how it contributes to hypoxemia is not known. Here we report that AKI induced rapid intravascular neutrophil retention in lung alveolar capillaries without extravasation into tissue or alveoli, causing hypoxemia by reducing lung capillary blood flow in the absence of substantial lung interstitial or alveolar edema. In contrast to direct ischemic lung injury, lung neutrophil recruitment during remote lung inflammation did not require cues from intravascular nonclassical monocytes or tissue-resident alveolar macrophages. Instead, lung neutrophil retention depended on the neutrophil chemoattractant CXCL2 released by activated classical monocytes. Comparative single-cell RNA-Seq analysis of direct and remote lung inflammation revealed that alveolar macrophages were highly activated and produced CXCL2 only in direct lung inflammation. Establishing a CXCL2 gradient into the alveolus by intratracheal CXCL2 administration during AKI-induced remote lung inflammation enabled neutrophils to extravasate. We thus discovered important differences in lung neutrophil recruitment in direct versus remote lung inflammation and identified lung capillary neutrophil retention that negatively affected oxygenation by causing a ventilation-perfusion mismatch as a driver of AKI-induced hypoxemia.

## Abstract 2

During sterile acute kidney injury CCR2^+ ^monocytes drive lung capillary neutrophil retention. The resultant reduced capillary perfusion induces a ventilation-perfusion deficit that causes hypoxemia.

## Graphical abstract

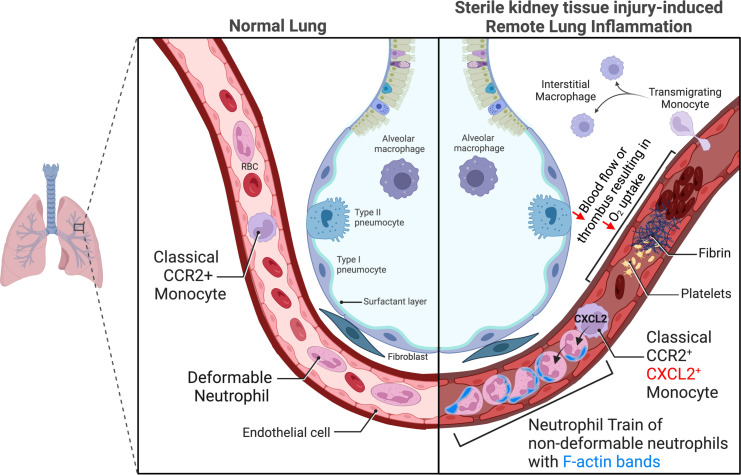

## Introduction

Sterile acute kidney injury (AKI) is a common clinical condition that carries a risk of high mortality, particularly when it occurs with respiratory failure (1–3). AKI-associated respiratory failure is incompletely understood and thought to result from lung interstitial and alveolar edema as a result of endothelial leakage in response to systemic inflammation induced by AKI (4, 5). In mice (4, 6–10) and humans (11, 12), AKI-induced systemic inflammatory mediators cause remote lung inflammation with neutrophil accumulation, but little to no alveolar inflammatory or serous exudates (reviewed in refs. 4, 7, 8). We showed that mice were hypoxemic 24 hours after AKI despite the lack of substantial alveolar edema or exudates and that prevention of lung neutrophil accumulation prevented hypoxemia (6). Which cellular cues attract neutrophils into the lung during remote inflammation and how neutrophils contribute to hypoxemia after AKI, however, remain largely unresolved.

Here we show that, during remote lung inflammation after AKI, the vast majority of neutrophils did not extravasate. Intravascular retention of neutrophils in pulmonary capillaries obstructed lung capillary blood flow and significantly reduced oxygenation by 2–6 hours after AKI in the absence of any marked interstitial or alveolar edema or inflammatory exudates. We show that, in remote lung inflammation, unlike in direct lung injury, nonclassical monocytes and alveolar macrophages were not required to attract neutrophils to the lung and, instead, implicated the neutrophil chemoattractant CXCL2 released by intravascular classical monocytes in capillary neutrophil retention. Comparative single-cell RNA-Seq (scRNA-Seq) analysis of direct and remote lung inflammation revealed that alveolar macrophages produced CXCL2 only during direct lung inflammation. Establishing a CXCL2 gradient into the alveolus by intratracheal (i.t.) administration of CXCL2 during AKI-induced remote lung inflammation enabled neutrophils to extravasate. We thus describe important differences in lung neutrophil recruitment in direct versus remote lung inflammation. We have uncovered what we believe to be a novel mechanism that underlies AKI-induced hypoxemia, namely intravascular neutrophilic inflammation with capillary perfusion deficits that worsen oxygenation despite proper alveolar function and ventilation. We hypothesize that lung intravascular neutrophil retention after AKI may protect the organism from overt systemic action of neutrophils and inflammatory mediators by sequestering them in the lung vasculature, yet at the risk of hypoxemia due to reduced lung alveolar capillary perfusion and oxygen uptake. Our findings affect the understanding of AKI-associated respiratory failure in general and also may help explain the negative effect AKI has on respiratory failure and mortality in patients with acute respiratory distress syndrome (ARDS) (13).

## Results

### Neutrophils recruited to the lung after AKI do not extravasate from lung vessels.

**Figure 1:**
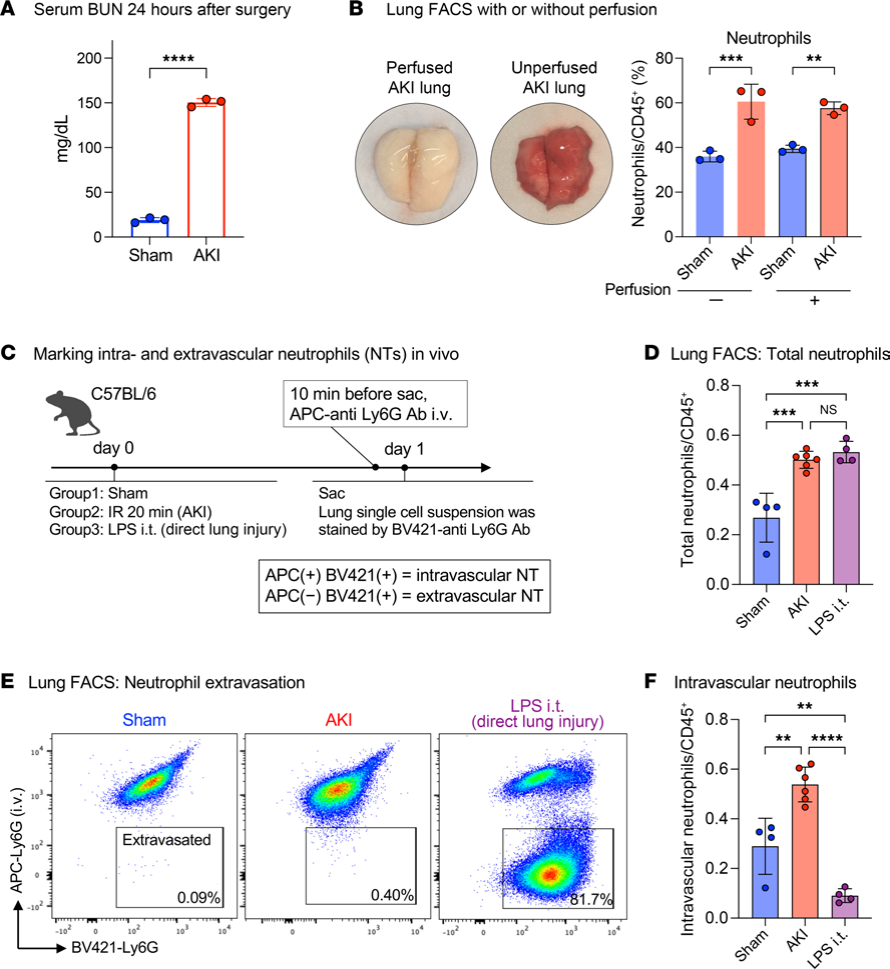
AKI induces intravascular neutrophil accumulation in the lungs. (**A**) Serum BUN levels 24 hours after sham or AKI surgery in WT mice. *n* = 3 per group. ****P < 0.0001, by unpaired, 2-tailed Student’s *t* test. (**B**) Representative image of mouse lung before and after perfusion with PBS. Total neutrophil counts in sham versus AKI lungs were quantified by flow cytometry (FACS) with or without prior lung perfusion. Neutrophils/CD45^+^ cells: nonperfused, 36.0% versus 60.5%; perfused, 39.4% versus 57.6%, in both sham versus AKI. *n* = 3 per group. **P < 0.01 and ***P < 0.001, by 2-way ANOVA with Šidák’s multiple-comparison test. (**C**) Schematic of the in vivo neutrophil extravasation assay using 2 different anti-Ly6G Abs coupled to different fluorescence labels (APC or BV421). Sac, sacrifice. (**D**) FACS quantification of lung total neutrophils after sham operation, AKI (remote lung inflammation), or i.t. LPS (direct lung injury). *n* = 4–6 per group. ***P < 0.001, by 1-way ANOVA with Tukey’s multiple-comparison test. (**E**) Identification of extravasated neutrophils by FACS. Ly6G fluorescence (APC vs. BV421) was analyzed in CD45^+^CD11b^+^Gr1^hi^ cells in sham-operated animals (left), AKI animals (middle), and animals subjected to direct lung injury by LPS i.t. (left). Single-positive (BV421^+^) cells are extravascular; double-positive cells (APC^+^BV421^+^) cells are intravascular. (**F**) FACS quantification of intravascular lung neutrophils (after PBS perfusion) for sham, AKI, or LPS i.t. *n* = 4–6 per group. **P < 0.01 and ****P < 0.0001, by 1-way ANOVA with Tukey’s multiple-comparison test. All data represent the mean ± SD.

During direct tissue injury, neutrophils typically establish inflammation by extravasating from the circulation into the injured tissue (14, 15). During remote lung inflammation after AKI, we and others have previously found that few, if any, neutrophils are detectable in bronchoalveolar lavage fluid (reviewed in refs. 4, 5, 16), indicating that they do not transmigrate through the pulmonary epithelial barrier. However, whether neutrophils exit the vascular space during remote lung inflammation is not well understood. We induced bilateral renal ischemia-reperfusion injury (IRI) in mice as a model of severe AKI. In WT mice, blood urea nitrogen (BUN) levels typically reach 100–150 mg/dL 24 hours after IRI (20 minutes of ischemia) (Figure 1A). Flow cytometric analysis confirmed a significant increase in the total number of lung neutrophils after AKI, irrespective of whether the lungs were perfused with PBS at the time of sample preparation (Figure 1B). This suggested that neutrophils either extravasated into the lung interstitium or that they could not be flushed out due to strong vascular adhesion or vascular obstruction. To determine the localization of neutrophils, we injected an allophycocyanin-labeled (APC-labeled) anti-Ly6G Ab i.v. 10 minutes prior to sacrifice, and a BV421-labeled anti-Ly6G Ab was added to lung single-cell suspensions generated from the same animal after sacrifice (17). Only intravascular neutrophils were double positive (APC^+^BV421^+^), while extravasated neutrophils were single positive (BV421^+^) (Figure 1C). We used direct lung injury by i.t. instillation of LPS as a positive control for neutrophil extravasation. Twenty-four hours after bilateral kidney IRI to induce AKI (ischemic AKI), the total number of neutrophils in the lung significantly increased over sham levels and was comparable to the increase observed after direct lung injury with LPS (Figure 1D). However, after AKI, more than 99.5% of neutrophils remained within the intravascular compartment, in contrast to direct lung injury, in which more than 80% of neutrophils had extravasated (Figure 1, E and F). Sterile kidney tissue injury–recruited lung neutrophils may therefore affect oxygenation by impeding blood flow in lung vessels. This is consistent with our published findings that AKI-induced remote lung inflammation with hypoxemia in our model lacks substantial alveolar edema or inflammatory exudates (6).

### Intravascular lung capillary neutrophil retention causes hypoxemia by reducing lung capillary perfusion.

**Figure 2:**
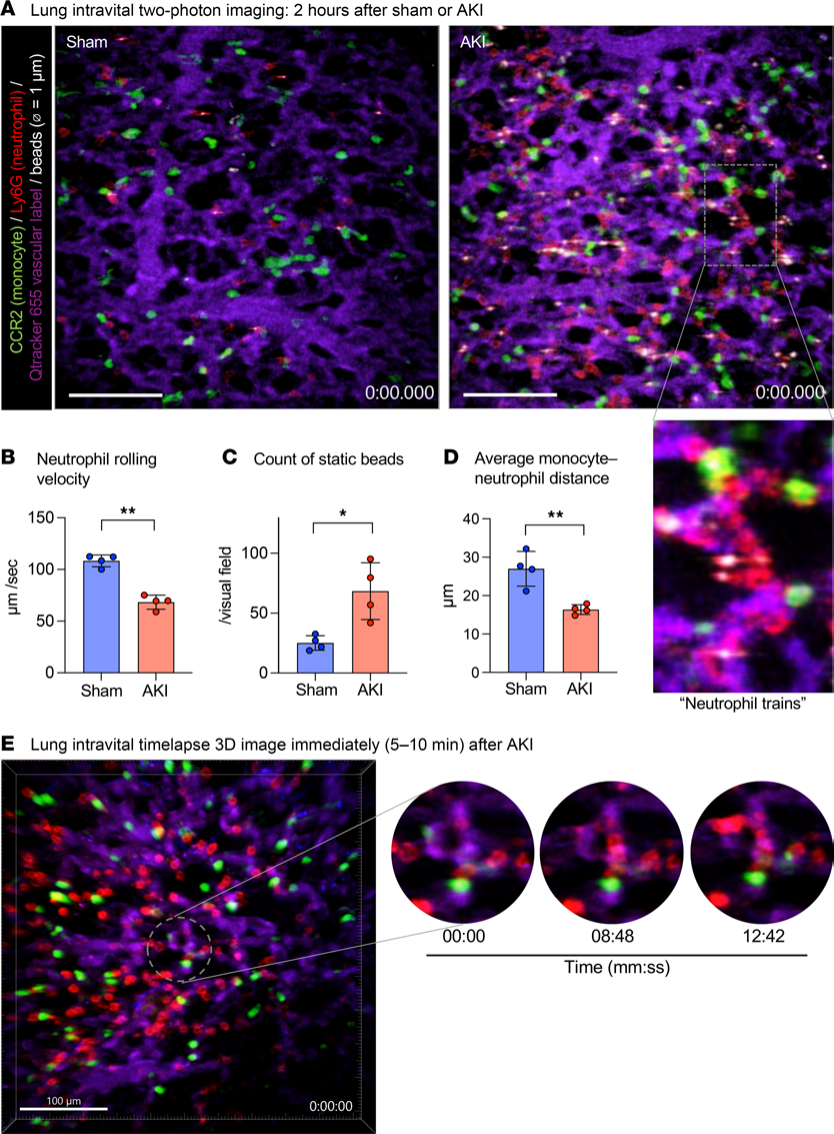
Intravascular lung capillary “neutrophil train” formation after AKI. (**A**) Intravital 2-photon imaging of sham-treated and AKI lungs 2 hours after AKI using *Ccr2^gfp/+^* mice and in vivo staining of neutrophils; CCR2^+^ monocytes (green), Ly6G^+^ neutrophils (red). Blood flow was assessed with 1 μm beads (white), and lung capillary circulation was labeled by i.v. injection of quantum dots (purple). The magnified inset image shows a neutrophil train in a lung capillary, illustrating vessel-occlusive accumulation of neutrophil trains attached to CCR2^+^ monocyte “locomotives.” Full videos are available in the supplemental materials. Scale bars: 100 μm; original magnification, x3.25 (inset). (**B**–**D**) Quantification of intravital imaging videos: speed of neutrophil rolling (**B**), the number of static beads (**C**), and the average distance between CCR2^+^ monocytes and neutrophils (**D**). *n* = 4 per group. *P < 0.05 and **P < 0.01, by unpaired, 2-tailed Student’s *t* test. (**E**) Lung intravital time-lapse 3D image immediately after AKI (5–10 minutes). Magnified images on the right illustrate the process of neutrophil (red) train formation in the presence of a CCR2^+^ monocyte (green). Scale bar: 100μm A full time-lapse video is available as Supplemental Video 3. Scale bar: 100 μm; original magnification, x3.25 (inset). All data represent the mean ± SD (**B**–**E**).

To determine early events that establish remote lung inflammation in real time, we investigated monocyte and neutrophil behavior in lungs using intravital 2-photon microscopy 2 hours after AKI as compared with uninjured sham-operated controls. We used *Ccr2^gfp/+^* mice that allow in vivo tracking of classical monocytes via GFP fluorescence (green) (18). Neutrophils, blood flow, and vessels were visualized in vivo by i.v. injection of a fluorescently labeled anti-Ly6G Ab (red), 1 μm fluorescent microbeads (white), and Qtracker655 (violet), respectively. Two hours after sham operation, few neutrophils, very few static microbeads, and few classical monocytes were detected in the lungs. Classical CCR2^+^ monocytes were largely localized at a significant distance from neutrophils (Supplemental Video 1; supplemental material available online with this article; https://doi.org/10.1172/JCI186705DS1). By contrast, 2 hours after AKI, we observed a large number of neutrophils lining up in lung capillaries that did not extravasate and formed neutrophil “trains,” which were generally tightly associated with at least 1 classical monocyte “locomotive” (see inset on right) (Figure 2A and Supplemental Video 2). Intravascular neutrophil speed was decreased to levels indicative of crawling/arrest (Figure 2B), and the presence of a large number of immobile intravascular 1 μm beads after AKI suggested severely reduced blood flow in the lung capillary microcirculation (Figure 2C). As additional supportive evidence for reduced capillary blood flow, we detected evidence of lung microthrombosis by antiplatelet (CD41a) and antifibrinogen immunofluorescence costaining (approximately 6% of the lung area) in AKI, but not sham-operated, lungs (Supplemental Figure 1). The average distance between classical monocytes and neutrophils was significantly reduced after AKI, raising the possibility of chemoattraction (Figure 2D). To detect neutrophil train formation and monocyte/neutrophil behavior at even earlier time points we obtained 3D time-lapse images of mouse lungs immediately after AKI (5–10 minutes after reperfusion) (Figure 2E and Supplemental Video 3). Already at this very early time point, we observed an increased number of neutrophils and CCR2^+^ classical monocytes in the lung. Overall monocyte speed was significantly reduced, with many monocytes not moving at all. At the same time, many neutrophils were already present in the lung, moving at much higher speeds than monocytes. In some instances, we could already observe the formation of neutrophil trains capped by a monocyte in lung capillaries. In such examples, neutrophils were moving toward an immobile monocyte, suggesting that the monocyte may send a neutrophil chemoattractant signal (Figure 2E). Of note, this intravascular neutrophil retention phenotype after AKI is very different from neutrophil behavior in direct lung injury due, for example, to warm lung IRI in a murine syngeneic lung transplant model (19). In direct lung injury, neutrophils do not remain in the intravascular space to form trains, but instead extravasate and form large alveolar swarms as early as 2 hours after reperfusion (Supplemental Video 4; Q-dot vascular dye: red; neutrophils: green). Taken together, our findings indicate that after AKI neutrophils accumulated very rapidly in lung capillaries, where they assembled into blood flow–impeding trains, possibly involving chemoattraction between neutrophils and monocytes.

**Figure 3:**
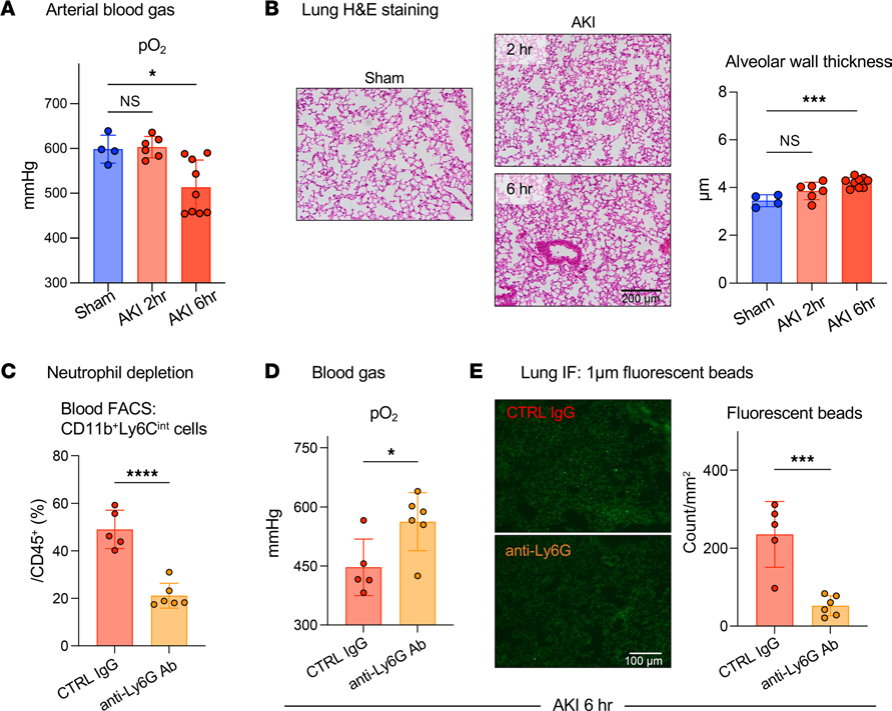
Hypoxemia is observed early after AKI in the absence of impaired ventilation or overt lung edema. (**A** and **B**) Arterial blood gas analysis and lung H&E staining with alveolar wall thickness measurements after sham operation or 2–6 hours after AKI. Arterial blood samples were collected directly from the ascending aorta under mechanical ventilation using 100% O_2_. Scale bar: 200 μm. *n* = 4–9 per group. ***P < 0.001, by 1-way ANOVA with Tukey’s multiple-comparison test. (**C**–**E**) Neutrophil depletion experiment. *n* = 5–6 per group. (**C**) Quantification of circulating neutrophils (CD11b^+^Ly6C^int^ cells) following 2 injections of anti-Ly6G Ab. Ly6G was not used as a neutrophil marker in FACS analysis due to epitope protection by the Ly6G Ab injected for neutrophil depletion. (**D**) Arterial blood gas analysis 6 hours after AKI. (**E**) Quantification of lung fluorescence beads injected 10 minutes prior to sacrifice as a surrogate marker for stagnation of lung microcirculation. Scale bar: 100 μm. *P < 0.05, ***P < 0.001, and ****P < 0.0001 compared with sham or control, by unpaired, 2-tailed Student’s *t* test. All data represent the mean ± SD.

We next asked whether the observed significant reduction in lung capillary blood flow could reduce blood oxygenation in the absence of substantial interstitial/alveolar edema or alveolar exudates (6). Compared with control conditions, arterial blood gas measurements revealed that, while no significant oxygenation defect was present at 2 hours, hypoxemia developed by 6 hours after AKI and correlated with the degree of kidney injury (Supplemental Figure 2). Oxygenation was reduced by approximately 16% at 6 hours (Figure 3A), similar to our previous observations at 24 hours after AKI in this model (6). No alveolar edema or exudates and only small alveolar wall thickness increases of 10%–15% were detectable at 2 and 6 hours after AKI (Figure 3B). Importantly, hypoxemia occurred between 2 and 6 hours after AKI without any substantial changes in alveolar wall thickness during the same time frame, implicating impaired capillary perfusion resulting in ventilation-perfusion deficits rather than interstitial edema as a cause of hypoxemia. Our findings are consistent with our previous report of only small alveolar wall thickness increases and absence of alveolar edema or exudates up to 24 hours after AKI, and with our previous finding that prevention of neutrophil accumulation during this time frame prevented hypoxemia (6). To further confirm that lung neutrophil accumulation is indispensable for impaired lung capillary perfusion and hypoxemia, we analyzed AKI animals following neutrophil depletion. Pretreatment with anti-Ly6G Ab effectively reduced the number of circulating neutrophils after AKI (Figure 3C), and neutrophil-depleted mice were protected from hypoxemia 6 hours after AKI (Figure 3D). The number of lung fluorescent microbeads i.v. injected 10 minutes before sacrifice was significantly higher in mice without neutrophil depletion (control IgG) than in those with neutrophil depletion (anti-Ly6G), suggesting that neutrophil retention in the lungs was indeed critical for the reduction in capillary flow and hypoxemia (Figure 3E).

### Rapid lung capillary neutrophil capture is enhanced by decreased neutrophil deformability but not classical neutrophil-endothelial cell interactions.

**Figure 4:**
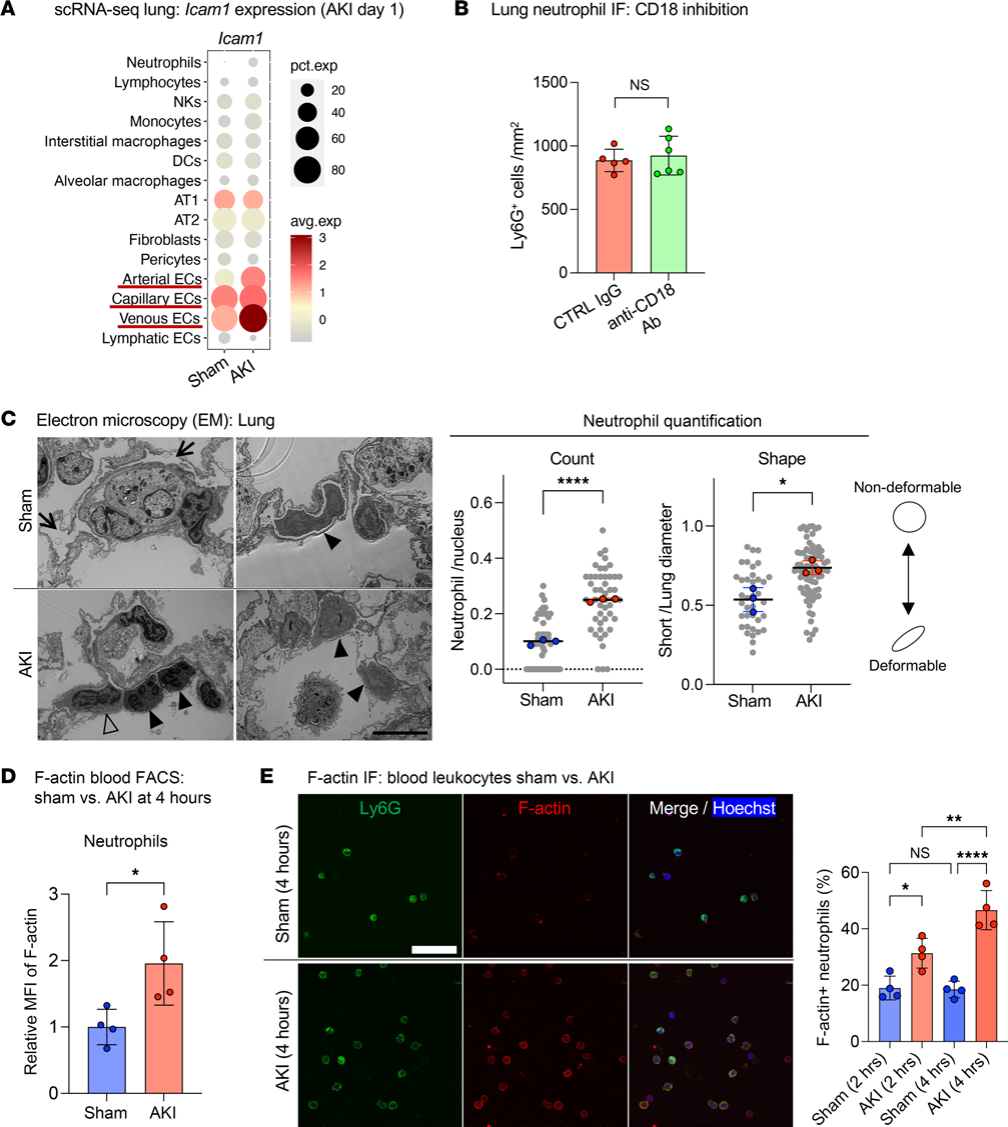
Rapid lung capillary neutrophil capture is enhanced by decreased neutrophil deformability but not classical neutrophil–endothelial cell interactions. (**A**) scRNA-Seq analysis (see Supplemental Figure 2) showing expression of the adhesion molecule ICAM1 in lung cell types. (**B**) Anti-CD18 Ab blockade versus IgG control in C57BL/6 mice: quantification of lung neutrophils by Ly6G immunofluorescence staining after AKI. *n* = 5–6/group. (**C**) Representative EM images of sham versus AKI lung obtained with quantification. Arrows indicate empty vessel lumina, while black arrowheads point to deformable neutrophils in sham or nondeformable neutrophils in AKI lung; in the AKI lung, an outlined arrowhead points to a monocyte locomotive leading a nondeformable neutrophil train. Right lower image shows crawling or arrested neutrophils (black arrowheads). Scale bar: 10 μm. Plot on the left shows the neutrophil count presented as the number of neutrophils per total nuclei in each image. Plot on the right shows the ratio of short versus long diameter in each neutrophil as an indicator of its morphology. The short/long ratio closer to 1.0 is indicative of a more rounded, nondeformable shape. Each colored dot represents mean data from an individual animal (*n* = 3/group), while each gray dot represents each observation (image or neutrophil). **P < 0.05 and ****P < 0.0001, by unpaired, 2-tailed Student’s t test (**B** and **C**). (**D**) Quantification of F-actin in circulating neutrophils by flow cytometry. Using fluorescence-labeled phalloidin, the geometric MFI was analyzed and compared between sham and AKI groups. *n* = 4/group. *P < 0.05, by unpaired, 2-tailed Student’s *t* test. (**E**) F-actin staining of leukocytes isolated from circulating blood 2 and 4 hours after sham or AKI. Scale bar: 40 μm. Percentage of F-actin polymerized Ly6G^+^ cells (neutrophils) are quantified in graph. *n* = 4/group. **P < 0.01 and ****P < 0.0001, by 2-way ANOVA with Šidák’s multiple-comparison test. All data represent the mean ± SEM.

Circulating neutrophils are attracted to sites of inflammation based on identifiable sequential interactions of different neutrophil cell-surface ligands with endothelial cell-surface receptors, encompassing canonical steps of neutrophil capture, rolling, crawling, arrest, and migration, prior to potential extravasation (20). Whether neutrophil–endothelial cell interactions are important in lung neutrophil recruitment after AKI is unknown. On the basis of our observations of neutrophil crawling/arrest during intravital imaging already 2 hours after AKI, we tested the blockade of key neutrophil–endothelial cell interactions that mediate neutrophil crawling/arrest and migration. The integrins LFA-1 (also known as CD11a/CD18) and Mac-1 (also known as CD11b/CD18) play overlapping roles in neutrophil arrest and migration (20, 21). Supporting the potential involvement of these integrins, in an analysis of scRNA-Seq datasets in sham and AKI mice intercellular adhesion molecule-1 (ICAM1), a ligand for LFA-1 or Mac-1, was found upregulated in lung endothelium on day 1 after AKI (Figure 4A). Supplemental Figure 3 provides a detailed description of our analysis using our previously published dataset (6), together with additional newly generated lung scRNA-Seq data. Despite endothelial cell upregulation of ICAM1, blockade of CD18 (Figure 4B) or LFA-1 (Supplemental Figure 4) did not reduce early neutrophil recruitment into the lungs 2 hours after AKI, suggesting that neutrophil accumulation during remote lung inflammation does not follow the classical neutrophil recruitment paradigm delineated largely in models of direct tissue injury (22).

We next hypothesized that rapid neutrophil capture in lung capillaries after sterile kidney tissue injury (<1 hour, as published by us in ref. 6 and beginning already 5–10 minutes after AKI, as shown in Figure 2E) may not depend on a specific capture signal that emanates from the lung. Instead, it could depend on reported changes in cellular deformability of primed neutrophils (23) stimulated by circulating inflammatory mediators, as has been described in humans with a systemic inflammatory response due to sepsis, trauma, or direct lung injury (24, 25). This is relevant because neutrophils have to be deformable to pass through the pulmonary microcirculation, as the average capillary diameter is approximately 7.5 μm (21, 26, 27), substantially smaller than neutrophils and monocytes, which have an average diameter of 9–15 μm (21). Nondeformability of primed neutrophils is conferred by rapid changes in their cellular shape induced by cytoskeletal rearrangement with the hallmark formation of polymerized F-actin bands in sub–cell membrane regions (28). We therefore used electron microscopy (EM) to examine cell shape and the presence of F-actin bands in neutrophils after AKI as compared with sham conditions. This analysis confirmed the intravascular neutrophil accumulation seen with intravital imaging. Neutrophilic cell shape was determined by measuring and comparing the ratio of the longest short to the longest long axis. Twenty-four hours after AKI, we detected mostly empty vessel lumina (arrows) and few neutrophils (black arrowheads) in sham lungs (Figure 4C, upper images and graph). Most neutrophils had a deformable, elongated cellular shape with a smooth cellular surface and no obvious interactions with the endothelium. Mice with AKI had an increased number of neutrophils within the vasculature (black arrowheads) that exhibited a less or nondeformable, rounded shape (Figure 4C, lower images and graph). As seen during intravital imaging, EM revealed monocytes (outlined arrowhead) in close association with neutrophil trains (black arrowheads) (Figure 4C left lower image). Next, we visualized F-actin polymerization and peripheralization using phalloidin staining in circulating neutrophils isolated from sham-operated or AKI mice. Flow cytometry detected significantly increased F-actin fluorescence in neutrophils 4 hours after AKI compared with sham treatment (Figure 4D). Immunofluorescence staining revealed that 20% of circulating neutrophils isolated from sham animals had sub–cell membrane F-actin bands, whereas in AKI animals, sub–cell membrane F-actin bands were detected in 30% of neutrophils at 2 hours, 40%–50% at 4 hours (Figure 4E), and 50% on day 1 after AKI (Supplemental Figure 5). Thus, the accumulation of neutrophils in lungs after AKI was likely significantly promoted by their priming and activation through kidney injury–released circulating factors that reduced their deformability, causing their rapid capture in pulmonary capillary vessels.

### Nonclassical monocytes or alveolar macrophages do not drive lung capillary neutrophil retention after AKI.

**Figure 5:**
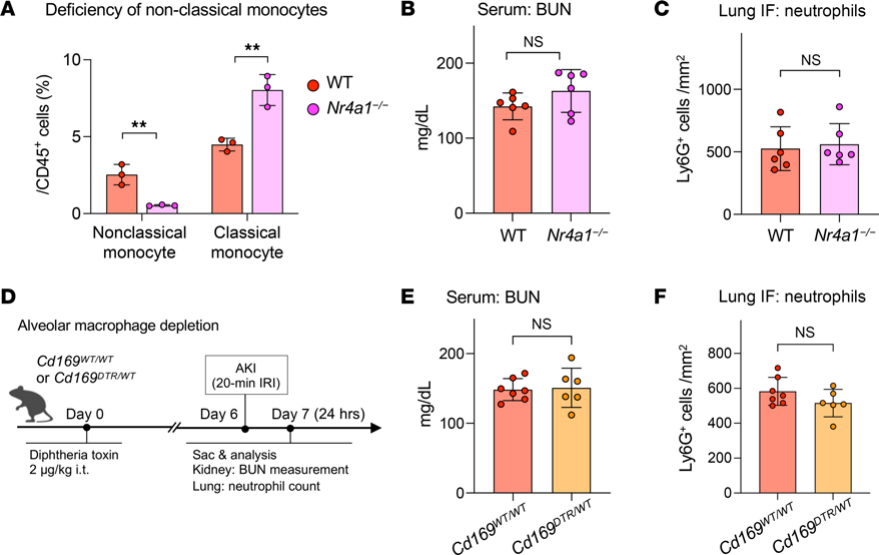
Neither nonclassical monocytes nor alveolar macrophages drive capillary neutrophil retention. (**A**) Quantification of blood monocytes in WT versus NR4A1-KO mice (*Nr4a1^–/–^*). *n* = 3 per group. **P < 0.01, by unpaired, 2-tailed Student’s *t* test. (**B**) Serum BUN levels 24 hours after AKI in WT and NR4A1-KO mice. *n* = 6 per group. (**C**) Quantification of lung neutrophils after AKI measured by immunofluorescence staining. *n* = 6 per group. (**D**) Schematic of in vivo alveolar macrophage depletion model using diphtheria toxin and CD169-DTR heterozygous mice. (**E**) Serum BUN levels 24 hours after AKI in CD169-DTR heterozygous mice and their littermate controls. *n* = 6 per group. (**F**) Quantification of lung neutrophils after AKI measured by immunofluorescence staining in CD169-DTR heterozygous mice and their littermate controls. *n* = 6 per group. Statistical significance in **B**, **C**, **E**, and **F** was determined by unpaired, 2-tailed Student’s *t* test. All data represent the mean ± SD.

We next aimed to elucidate which cellular cues are needed for AKI-induced lung neutrophil accumulation. In direct lung injury, neutrophil recruitment and extravasation is driven by cues derived from nonclassical monocytes (29), alveolar macrophages (30), and classical monocytes (31). *Nr4a1^–/–^* mice (32) lack nonclassical monocytes but not classical monocytes, as shown by Ly6C flow cytometry of circulating monocytes. Classical monocytes in *Nr4a1^–/–^* mice were in fact a little elevated because they could not transition into nonclassical monocytes (Figure 5A). *Nr4a1^–/–^* mice experienced kidney injury in response to bilateral IRI similar to that of the WT controls (Figure 5B) but showed no differences in lung neutrophil accumulation after AKI (Figure 5C). We next eliminated alveolar macrophages with i.t. diphtheria toxin (DTX) application in *Cd169^DTR/+^* mice (Figure 5D Experimental Scheme and Supplemental Figure 6). *Cd169^DTR/+^* mice treated with DTX showed kidney injury comparable to that in WT controls (Figure 5E), but alveolar macrophage depletion did not affect lung neutrophil accumulation after AKI (Figure 5F). Thus, nonclassical monocytes and alveolar macrophages did not play critical roles in mediating neutrophil accumulation in lungs after AKI.

### CCR2^+^ classical monocytes and CXCL2 drive lung capillary neutrophil retention.

**Figure 6:**
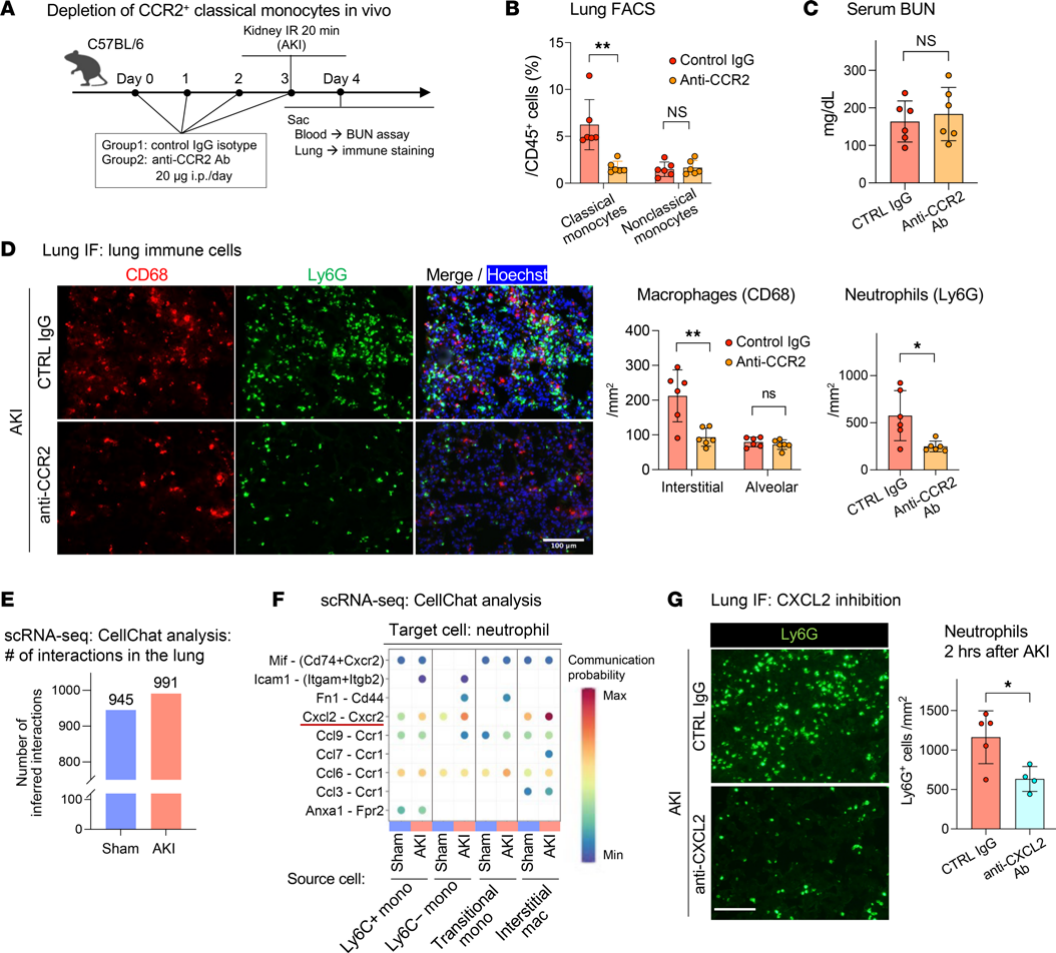
CCR2^+^ classical monocytes and CXCL2/CXCR2 signaling drive lung capillary neutrophil retention after AKI. (**A**) Schematic of classical monocyte depletion using anti-CCR2 Ab in vivo. (**B**) Lung monocytes after pretreatment with anti-CCR2 Ab or control IgG. *n* = 6 per group. (**C**) Serum BUN indicating kidney injury level on day 1 after AKI. *n* = 6 per group. (**D**) Lung immunofluorescence staining after AKI in control versus anti-CCR2 Ab–treated animals. Shown are alveolar and interstitial macrophages (CD68^+^, red) and neutrophils (Ly6G^+^, green). Hoechst 33342 dye (blue) was used to visualize nuclei. Scale bar: 100 μm. *n* = 6 per group. (**E**) Total number of cell-cell communications inferred by CellChat based on the scRNA-Seq analysis for sham-operated versus AKI mice (also see Supplemental Figure 2). (**F**) Cell-cell communications from monocytes or macrophages to neutrophils were predicted at single ligand-receptor resolution (unbiased); the neutrophil chemoattractant CXCL2 in monocytes and macrophages and their receptor CXCR2 in neutrophils was predicted to be significantly increased in AKI versus sham groups and are underlined. (**G**) Lung immunofluorescence staining for Ly6G^+^ neutrophils (green) from WT C57BL/6 mice injected with anti-CXCL2 or control Ab and subjected to AKI. Scale bar: 100 μm. *n* = 4–5 per group. The graph shows a quantification of lung neutrophils detected by immunofluorescence. Data represent the mean ± SD. *P < 0.05 and **P < 0.01, by unpaired, 2-tailed Student’s *t* test.

Since classical monocytes appeared to interact with neutrophils, as shown by intravital imaging and EM in lung capillaries, we examined their role in establishing lung capillary neutrophil trains. First, we selectively depleted CCR2^+^ classical monocytes by administration of anti-CCR2 Ab (33) (Figure 6A). This Ab does not deplete circulating neutrophils (34). Anti-CCR2 Ab pretreatment depleted classical monocytes (CD11b^+^, Ly6G^–^, CCR2^+^, Ly6C^+^), but not nonclassical monocytes (CD11b^+^, Ly6G^–^, Ly6C^–^) (Figure 6B). We observed no differences in kidney injury between anti-CCR2 or control Ab–treated animals (Figure 6C). However, depletion of classical monocytes inhibited lung neutrophil and also interstitial macrophage accumulation after AKI almost completely as compared with the control (Figure 6D). These results identified classical CCR2^+^ monocytes as key upstream players in AKI-induced lung neutrophil accumulation.

To identify possible immune cell signals that recruit neutrophils, in particular emanating from classical CCR2^+^ monocytes, we performed cell-cell communication analysis with CellChat, a software platform that predicts intercellular communication networks on the basis of expression data of known ligand-receptor pairs. CellChat analysis applied to a lung scRNA-Seq dataset from sham-operated and AKI mice (Supplemental Figure 3) predicted neutrophil chemoattractant signals that may drive neutrophil train formation after AKI. The total number of predicted signaling interactions in the lung vascular niche between neutrophils, monocytes, transitional monocytes, interstitial macrophages, and endothelial cells modestly increased after AKI (~5%) (Figure 6E). Cell-cell communication analysis comparing the sham control with AKI predicted a strong relevance of classical monocyte-to-neutrophil signaling after AKI via action of the neutrophil chemoattractant CXCL2 on the CXCR2 receptor expressed in neutrophils (Figure 6F). It also predicted that CXCL2 produced by neutrophils acts on neutrophils themselves in an autocrine loop. Injection of a CXCL2-neutralizing Ab into AKI animals as compared with the IgG control significantly suppressed neutrophil train formation in the lungs (Figure 6G). These data suggest that CXCL2 released intravascularly by CCR2^+^ classical monocytes established a critical CXCL2 gradient within lung capillary vessels that was required to form neutrophil trains by attracting neutrophils to monocytes. This was further supported by our intravital imaging findings at very early time points after AKI (Figure 2E).

### Comparative analysis of lung scRNA-Seq data reveals that remote lung injury as compared with direct lung injury lacks alveolar macrophage activation and their release of neutrophil chemoattractants.

To discern differences between direct and remote lung inflammation after AKI that may explain neutrophil extravasation and transmigration into alveoli in the former but not in the latter, we performed comparative scRNA-Seq analysis of the transcriptomic landscape of lungs from control and syngeneic lung transplants, representing a model of direct lung injury (warm IRI) with high neutrophil extravasation and transmigration into alveoli (35), and of lungs from sham-operated or AKI-treated animals, notably lacking extravasation and transmigration into alveoli (Figure 1). We scaled, standardized, and integrated scRNA-Seq data from mouse control lungs and syngeneic lung transplants 2 hours after transplantation (36), together with our aforementioned lung scRNA-Seq dataset from sham-treated and AKI mice. After excluding doublets and cells with low-quality expression data, 73,814 lung cells underwent clustering using Seurat, version 4 (37).

**Figure 7:**
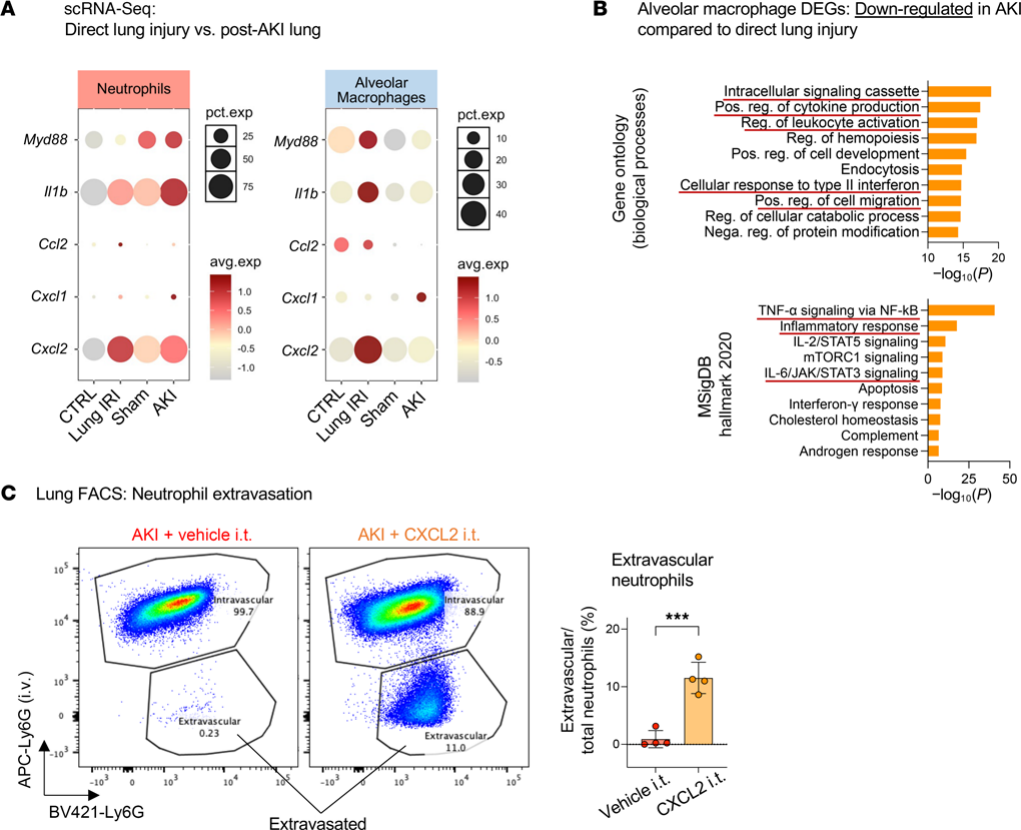
Comparative analysis of lung scRNA-Seq data reveals that remote lung injury as compared with direct lung injury lacks alveolar macrophage activation and alveolar macrophage release of neutrophil chemoattractants. (**A**) Comparison of inflammatory genes using scRNA-Seq analysis. A lung syngeneic transplant dataset was analyzed as a model of direct lung injury and compared with our AKI dataset (both reflect warm IRI). Expression levels of inflammatory molecules in neutrophils and alveolar macrophages are shown. The dot size denotes the percentage of cells expressing each gene, and the color scale represents the average gene expression levels. CTRL, control. (**B**) DEG analysis in alveolar macrophages: downregulated genes in post-AKI lung compared with direct lung injury were analyzed using GO and MSigDB Hallmark pathway analysis. GO terms and pathways related to inflammation and associated with activation of macrophages are underlined. Nega. reg., negative regulation; Pos. reg., positive regulation. (**C**) FACS and quantification of extravasated neutrophils 24 hours after AKI. Either mouse CXCL2 protein (0.01 μg/g) or vehicle in 50 μL sterile saline was administered i.t. immediately after AKI surgery. *n* = 4 mice per group. ***P < 0.001, by unpaired, 2-tailed Student’s *t* test. Data represent the mean ± SD.

**Figure 8:**
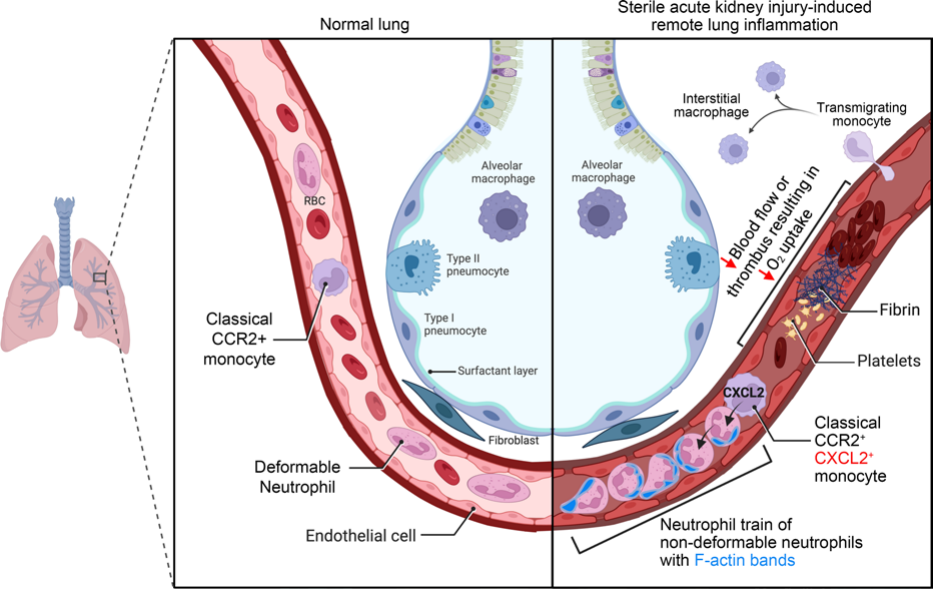
Schematic summary. AKI induces rapid intravascular neutrophil train formation in lung alveolar capillaries, a form of neutrophil retention. Rapid retention is enhanced by decreased deformability secondary to F-actin polymerization (submembrane F-actin bands) in circulating neutrophils that impedes their lung capillary passage. CCR2^+^ classical monocytes are required for neutrophil train formation and release CXCL2 to attract neutrophils into trains. Neutrophil train formation reduces alveolar capillary blood flow and is associated with thrombosis (thrombi contain both platelets and fibrin). This capillary perfusion defect leads to reduced oxygenation due to a ventilation perfusion mismatch, a scenario that differs from infectious inflammatory lung diseases, such as bacterial pneumonia or pulmonary alveolar edema in which ventilation is affected.

When comparing scRNA-Seq data of direct lung injury with data of AKI-induced remote lung inflammation relative to respective controls, we found that neutrophils in remote lung inflammation (exclusively intravascular) compared with neutrophils in direct lung inflammation (mostly extravasated) showed much higher innate immune signaling with upregulation of *Myd88*, a downstream effector of signaling via TLRs in response to damage-associated molecular patterns (DAMPs) (38), and of *IL1b*, a major effector of early type 1 immune responses (39). In contrast, alveolar macrophages in direct, but not in remote, lung inflammation showed substantial activation of innate immune signaling, again with *Myd88* and *Il1b* upregulation, and strong expression of the neutrophil chemoattractant CXCL2 (Figure 7A). Consistent with this, analysis of differentially expressed genes (DEGs) showed that alveolar macrophages had less leukocyte activation and cytokine signaling after AKI compared with direct lung injury (Figure 7B). A comprehensive comparison of monocyte/macrophage populations in direct and remote lung inflammation also highlighted the lack of alveolar macrophage activation after AKI, whereas other cell types (interstitial macrophages and Ly6C^−^ and Ly6C^+^ monocytes) exhibited comparable or even more intense transcriptomic inflammatory signatures after AKI than after direct lung injury (Supplemental Figure 7). CXCL2 production by alveolar macrophages could establish a CXCL2 gradient directed toward the alveolus that may have been responsible for the observed neutrophil extravasation and transmigration into alveoli in direct lung injury. Furthermore, our analysis suggests that this alveolar macrophage-derived extravasation signal was missing in AKI-induced remote lung inflammation. To assess whether neutrophils are, in principle, able to extravasate during AKI-induced remote lung inflammation, we instilled CXCL2 i.t. just after sham or AKI. In comparison with vehicle control a one-time instillation of CXCL2 into the alveolus right after AKI significantly increased the rate of neutrophil extravasation by over 20-fold at 24 hours (0.41% to 11%) (Figure 7C). Alveolar CXCL2 was able to induce extravasation of comparably more intravascular neutrophils in sham mice than in AKI mice (Supplemental Figure 8), indicating that neutrophil stiffness, which was more prominent in AKI neutrophils (Figure 4), at least partially contributed to the neutrophil intravascular retention phenotype. These findings suggest that the absence of alveolar macrophage activation and the lack of a neutrophil chemoattractant gradient into the alveolus probably contributed to the relative lack of neutrophil extravasation after AKI. A summary of our findings is shown in Figure 8.

## Discussion

Our work reveals that lung neutrophil recruitment after sterile kidney tissue injury as compared with direct lung injury required different cellular cues, localized almost exclusively to the intravascular compartment, and was mechanistically linked to the development of hypoxemia by causing a lung capillary perfusion defect, rather than by affecting alveolar function and ventilation.

A role of monocytes and alveolar macrophages in lung neutrophil recruitment and their extravasation different from sterile AKI has been demonstrated in direct lung injury after bacterial challenge and during lung IRI, a process inherent to pulmonary transplantation (35). Here, intravascular lung nonclassical monocytes were activated by lung tissue injury–released DAMPs and drove neutrophil accumulation in lung vessels by production of the neutrophil chemoattractants CXCL1 and CXCL2 (19, 29). At the same time, nonclassical monocytes produced IL-1β, which activates tissue-resident alveolar macrophages to produce the monocyte chemoattractant CCL2. CCL2 recruits CCR2^+^ classical monocytes into lung vessels, a requirement for the observed massive neutrophil extravasation into lung tissue and alveoli during direct injury (30; reviewed in ref. 40). In contrast, our results show that nonclassical monocytes and alveolar macrophages were dispensable for AKI-induced remote lung neutrophil accumulation. Alveolar macrophages did not show activation of innate immune signaling pathways during AKI, as opposed to direct lung injury, suggesting that AKI-induced innate immune–activating signals from the circulation were unable or not strong enough to reach alveolar macrophages. However, alveolar macrophages do play a role in sepsis-induced remote lung inflammation, another form of indirect lung injury. In sepsis from extrapulmonary infections, alveolar macrophages become activated and drive significant neutrophil extravasation into the alveolar space (41). Such alveolar neutrophil extravasation is in fact also observed in septic patients with ARDS (septic ARDS) (42; reviewed in ref. 43), suggesting that sepsis induces additional signals that reach and activate alveolar macrophages or that during sepsis, intravascular inflammatory and innate immune–activating signals are more pronounced than during AKI. Mimicking a neutrophil chemoattractant signal emanating from the alveolus, i.t. instillation of the neutrophil chemoattractant CXCL2 induced extravasation of neutrophils after AKI, and even better after sham surgery. This shows that the nondeformability of neutrophils did not principally interfere with extravasation, but that better deformability, as seen in sham procedure neutrophils, enhanced extravasation in the presence of an alveolar chemoattractant signal. Thus, neutrophils had the capacity to extravasate after AKI but lacked the alveolar signals to do so. CCR2^+^ monocytes are drivers of neutrophil extravasation into injured tissues not only in direct lung injury (31), but also, for example, during arthritis, as shown with intravital imaging (34). We can only speculate why CCR2^+^ monocytes do not have this function in remote lung inflammation. Perhaps alveolar macrophage activation is necessary, in addition to CCR2^+^ monocyte activation, to mediate neutrophil extravasation, as is observed in direct lung injury.

We and others have previously reported on the lack of neutrophil transmigration into alveoli up to 24 hours after AKI, based on bronchoalveolar lavage analysis in mice (6, 44). This suggested that neutrophils may not extravasate and/or transmigrate through the pulmonary epithelial barrier during remote lung inflammation, in mice or humans. Enhanced neutrophil accumulation in lung vessels 24 hours after AKI in mice has been observed, but lung tissue validation was limited, and its physiological significance was not studied (9). The normal lung circulation is unique, in that it contains marginated intravascular neutrophils and also classical and nonclassical monocytes, which together form an immune niche poised to rapidly respond to various immune challenges, in particular invasion of pathogens (45). The entire cardiac output passes through the lungs, such that during dissemination of infection via the bloodstream pathogens have to pass through the lungs. Like sepsis, AKI induces a systemic inflammatory response syndrome (SIRS) involving many of the same circulating proinflammatory mediators and also an increase in circulating neutrophils and monocytes (7). Pathogens in the bloodstream induce the formation of sizable intravascular neutrophil swarms in larger-caliber lung vessels that facilitate pathogen capture and clearance and serve as an important evolutionarily conserved defense mechanism against sepsis (46). Using intravital lung imaging, we show that AKI induced a different form of intravascular neutrophil accumulation, namely intravascular neutrophil retention in neutrophil trains. Lung neutrophil trains impeded blood flow in alveolar capillaries, sites where an intact circulation is required for oxygen exchange. Our work reveals that capillary neutrophil trains were able to reduce capillary blood flow to a degree that rapidly affected oxygenation after AKI (within 2–6 hours), even without marked interstitial or alveolar edema and alveolar exudates. Based on our previous observation that lung neutrophil accumulation reaches a plateau 12–24 hours after AKI (6), neutrophil train formation and lung capillary flow impediment likely progressed significantly after the 2-hour intravital imaging time point we obtained (Figure 2A) but became critical for oxygenation somewhere between 2 and 6 hours after AKI. A possible correlate of reduced capillary blood flow, AKI-induced RBC “rouleaux” formation in lung vessels has been reported previously, but its effects on blood flow or physiological outcomes was not determined (47). We speculate that lung capillary intravascular neutrophil train formation and slowed lung capillary blood flow after sterile kidney tissue injury represent an adaptive reaction that may serve to sequester activated immune cells and circulating cytokines in the lung, protecting the organism from their overt systemic action, but at the expense of causing blood oxygenation deficits that may result in hypoxemia (maladaptive consequence). Extensive sepsis-induced neutrophil swarms can indeed also negatively affect oxygenation (48). Widespread medium-to-large vessel arterial and venous thrombosis containing neutrophil macroaggregates has been described after gut ischemia in mice and also in patients with severe ARDS (49).

We show that rapid lung neutrophil capture after AKI was enhanced by an induced reduction in neutrophil deformability. Changes in neutrophil deformability (23) and lung retention lasting more than 40 minutes have been observed in humans following the injection of ex vivo primed neutrophils (50), and changes in flow and adhesive properties (“stiffening”) of circulating immune cells have also been documented in humans with trauma (24), ARDS (51), cardiac tissue injury (52), or sepsis (53, 54). Canonical steps of neutrophil recruitment that mediate crawling and arrest, such as LFA-1/ICAM1 or Mac-1/ICAM1 neutrophil–endothelial cell interactions, are not required in remote lung inflammation. This is not unprecedented, as neutrophil recruitment is strongly context dependent (46) and does not always involve integrin activation, and noncanonical pathways involved in neutrophil recruitment have been described in different tissues (55).

In several contexts, leukotriene B-4 (LTB-4) is required for the initiation of intravascular neutrophil swarming and clustering behavior (reviewed in ref. 46), in particular in fungal sepsis (48). However, we did not find any significant expression of LTB-4 receptors in lung cells after sterile kidney tissue injury. Instead, our model shows the involvement of the neutrophil chemoattractant CXCL2 released by classical monocytes as the signal that assembles captured neutrophils into trains in the lung early after AKI. At later time points, as our lung cell-cell communication analysis on day 1 after AKI suggests, neutrophils produce CXCL2 themselves, likely contributing to increased train length and their retention in the lung. CXCL2 has not been directly linked to lung neutrophil recruitment after AKI, but mice globally deficient in the CXCL1/2 receptor CXCR2 show reduced remote lung neutrophil recruitment after AKI (56), supporting our data. Other source cells and lung neutrophil chemoattractants in AKI-induced remote lung inflammation have been identified. For example, neutrophil accumulation has also been linked to AKI-induced CXCL1 produced by lung endothelial cells. Inhibition of IL-6 in WT mice reduced remote lung inflammation, lung CXCL1 expression, and lung neutrophil accumulation after AKI (10). IL-6 injection into IL-6–deficient mice with AKI induces lung CXCL1 expression and lung neutrophil accumulation; CXCL1-neutralizing Ab or CXCR2 global KO confers protection (56). Lung CXCL1 protein colocalizes with endothelial cell markers in lung tissue, suggesting that CXCL1 may be produced by endothelial cells after AKI (56). Lung neutrophil recruitment driven by endothelial CXCL1 may act in concert with the CXCL2 mechanism described in the present work. However, our scRNA-Seq analysis 24 hours after AKI compared with sham controls did not reveal significant CXCL1/2 expression in lung endothelial cells (6).

While lung edema can occur after AKI due to volume overload with left ventricular failure in the setting of oliguria (cardiogenic edema), patients with AKI with respiratory compromise frequently do not show signs of volume overload such as elevated pulmonary artery wedge pressure on cardiac catheterization (57, 58), and also do not significantly improve after volume removal using dialysis (12, 59). Instead, noncardiogenic lung edema driven by endothelial injury in response to AKI-released circulating inflammatory factors resulting in endothelial leak with interstitial and alveolar edema has been proposed as the dominant mechanism of AKI-induced hypoxemia. Interstitial wall thickness increases, and alveolar edema would interfere with ventilation and oxygenation by increasing the distance between the ventilated space and RBCs traveling in capillaries (reviewed in refs. 4, 5, 16). Our findings provide an alternative, or at least additional, explanation for respiratory failure in the context of AKI that does not improve with fluid removal, whether AKI occurs in isolation or as part of existing respiratory failure from primary or secondary causes (such as COVID-19, bacterial pneumonia, sepsis, or trauma). The lack of substantial interstitial/alveolar edema and of inflammatory alveolar exudates after AKI likely explains why such patients frequently show only subtle changes on their chest radiographs that cannot explain the observed degree of hypoxemia. Strategies to detect and prevent remote lung inflammation and capillary neutrophil train formation may represent therapeutic avenues to diminish or prevent AKI-induced respiratory complications in the clinic. Since the observed lung effects develop rapidly and AKI is often detected late in the clinic, how neutrophil trains can be detected, deprimed, dissolved, and released from the lungs appear particularly important points to address in future research in critically ill patients with AKI.

## Methods

### Sex as a biological variable.

Our study used male C57BL/6 mice because female mice exhibit high resistance to ischemic injury, have high mortality, and show large variability in the measurements used in this study. However, we have also observed the kidney-lung phenotype we describe here in female mice. We thus expect the findings in male mice presented here to be relevant for both sexes.

### Animal experiments.

Eight- to 12-week-old male C57BL/6 (strain 000664) and NR4A1-KO (strain 006187) mice were purchased from The Jackson Laboratory and kept on a 12-hour light/12-hour dark cycle with liberal access to water and a standard chow diet. In our AKI model, 20 minutes of bilateral IRI was introduced at 37°C using the flank approach as previously described (6). Sham operation was performed using the same surgical procedure without clamping the renal hilum with surgical clips. In select experiments, following intubation with a 20 gauge catheter, 0.01 μg/g LPS derived from *Pseudomonas aeruginosa* (MilliporeSigma, L8643) or 0.01 μg/g mouse CXCL2 (R&D Systems, 452-M2-010) dissolved in 50 μL sterile saline was i.t. instilled. For the neutrophil depletion experiment, mice were treated with 400 μg anti-Ly6G Ab (Bio X Cell, BE0075) or the same amount of rat IgG isotype control Ab 24 hours before and immediately before AKI surgery. For CCR2^+^ monocyte depletion, C57BL/6 mice were injected with anti-CCR2 Ab (MS-21) at 20 μg per day for 4 consecutive days (33, 60) prior to IR injury. For CD18 and CXCL2 inhibition, 200 μg anti-CD18 (Bio X Cell, BE0009) and 100 μg anti-CXCL2 mAb (Invitrogen, Thermo Fisher Scientific, MA5-23737) were injected, respectively (half the dose i.p. 1 hour prior to ischemia and the other half i.v. at reperfusion). The control animals were treated with the same dosage of rat IgG isotype (Invitrogen, Thermo Fisher Scientific, 10700). The organs were harvested 2–24 hours after IR injury, as specified in each experiment. In the blood gas measurement experiment, mice were intubated following injections of ketamine (90 μg/g) and xylazine (10 μg/g) and then mechanically ventilated with 100% O_2_. Arterial blood samples were directly collected from the ascending aorta using a syringe with a 30 gauge needle under a surgical microscope. These samples were analyzed within 1 minute using the i-STAT portable blood gas analyzer system (Abbott).

### Single-cell preparation from mouse lung.

Twenty-four hours after sham or AKI surgery, mice were euthanized and perfused with 20 mL ice-cold PBS from the left and right ventricles to remove remaining blood in the lung vasculatures. Perfused lung tissue was immediately dissected and chopped into pieces smaller than 1 mm^3^ by scalpels and razors. For single-cell preparation, samples were incubated for 60 minutes at 37°C with gentle shaking in RPMI media supplemented with 50 mg/mL Liberase TL (Roche, 5401020001), 40 U/mL DNAse I (MilliporeSigma, D4527), and 0.75 mg/mL hyaluronidase (MilliporeSigma, H3506). At the end of incubation, each sample was filtered twice, once through a 70 mm and once through a 40 mm strainer. RBCs in the samples were removed by ACK lysing buffer (Quality Biological, 118156101). Single-cell suspensions were then washed and resuspended in FACS buffer (PBS, 0.5% BSA, 2 mM EDTA, 0.01% NaN_3_)and used for scRNA-Seq and/or flow cytometric analysis.

### scRNA-Seq and data analysis.

scRNA-Seq analysis was conducted on lung and kidney samples from sham-treated and AKI mice as previously described (6). Briefly, a total of 6 samples were pooled for each group, and live cells were sorted using propidium iodide staining with a FACSAria III (BD Biosciences) flow cytometer. cDNA libraries were prepared using the Chromium Single Cell 5′ Library Kit, version 2 (10X Genomics) following the manufacturer’s instructions. The amplified full-length cDNA libraries were then submitted to the Genome Technology Access Center of Washington University for sequencing, with a sequencing depth of 50,000 reads. All subsequent data processing steps were performed using R software, version 4.1.0, with Seurat, version 4 (37). Expression matrices for each sample were imported into R as Seurat objects. Quality control was initially performed by retaining cells with gene expression counts ranging from 200 to 3,200. Cells with more than 10% mitochondrial genes and genes not expressed in at least 3 cells were excluded. After these quality control steps, the SCTransform and Harmony functions were applied for normalization, scaling, variance stabilization, and integration of the datasets (61, 62). After quality control and integration, clustering was done by applying a K-nearest neighbor graph with a resolution of 0.2 on the result of principal component analysis (PCA). Clustering was visualized using uniform manifold approximation and projection (UMAP). To assign cluster identities, a list of lung and kidney cell types and their currently established markers was first compiled (63, 64), and manual annotation was performed using those markers and additional genes with differential expression between clusters identified by the FindAllMarkers() function of Seurat. The expression levels of genes of interest in each cluster were visualized using the plot1cell package (65). Cell-cell communication analysis within the scRNA-Seq datasets was performed using CellChat (66). The functional properties of statistically significant DEGs (*P <* 0.05) were then investigated by gene ontology (GO) term analysis using Metascape (67) and MSigDB Hallmark 2020 Pathway analysis with Enrichr (68).

### Flow cytometric analysis.

Single-cell suspensions were stained with LIVE/DEAD Aqua cell dye (Invitrogen, Thermo Fisher Scientific, L34965, dilution 1:200) for 30 minutes. After washing with PBS, FcR blocking with anti–mouse CD16/32 Ab (BioLegend, 101302, 1:100) was performed for 15 minutes in FACS buffer, followed by a 30-minute incubation with the following diluted Abs: BV605 anti–mouse CD45 (BioLegend, 103139, 1:200); BV421 anti–mouse Ly6G (BioLegend, 127627, 1:200); BUV395 anti–mouse CD11b (BD Biosciences, 563553, 1:200); BV786 anti–mouse SiglecF (BD Biosciences, 740956, 1:200); FITC anti–mouse F4/80 (Invitrogen, Thermo Fisher Scientific, 11480181, 1:100); PE anti–mouse MERTK (BioLegend, 151506, 1:100); and APC anti–mouse Ly6C (BioLegend, 128016, 1:400). Unstained and single stained compensation samples were prepared from live lung or splenic cells for each FACS run. For quantification of F-actin in neutrophils, blood leukocytes were incubated with rhodamine-conjugated phalloidin (Invitrogen, Thermo Fisher Scientific, R415, 1:200) for 30 minutes at room temperature after permeabilization by 0.2% Tween 20. FACS analysis was performed on a BD Fortessa X20 with 5 lasers, and data were analyzed with FlowJo software (BD).

### In vivo neutrophil extravasation assay.

In order to distinguish intravascular from extravasated neutrophils, 10 μL APC anti–mouse Ly6G Ab (BioLegend, 127614) was i.v. injected into mice 10 minutes before sacrifice, as previously reported (17). After dissociation of lungs into single-cell suspensions, each sample was stained by another anti–mouse Ly6G Ab (BioLegend, 127627) carrying a different fluorescence (BV421). During FACS analysis, intravascular neutrophils were identified as double positive (APC^+^BV421^+^), whereas extravasated neutrophils were single positive (APC^+^BV421^−^), since they were not reached by the first Ab that had been i.v. applied.

### Intravital imaging of the lungs.

Intravital lung imaging in CCR2-GFP mice was performed using 2-photon microscopy as previously described (35). Briefly, 1.5 hours after IR or sham surgery, mice were orotracheally intubated with a 20 gauge catheter and ventilated with 1.5% isoflurane in air supplemented with 2 L/min oxygen (tidal volume: 220–250 μL, respiratory rate 250/min). To visualize neutrophils after AKI or sham treatment, blood vessels, and blood flow, 8 μL PE anti–mouse Ly6G Ab (BioLegend, 127608), 10 μL of 655 nm Q-dots (Invitrogen, Thermo Fisher Scientific, Q21021MP), and 5 μL of 1 μm fluorescence beads (Invitrogen, Thermo Fisher Scientific, F13080) were i.v. injected prior to imaging. A thoracotomy with partial removal of 4–5 left lower ribs exposed enough lung tissue for 2-photon microscope imaging. Our strategy allowed intravital lung imaging approximately 2 hours after sham treatment or AKI. The video rate was set to 300 milliseconds, and 6 random areas of the lung were recorded for at least 25 seconds per animal. The entire surgical and acquisition process was conducted on a heated pad to maintain the body temperature of the mice at 37°C. To obtain intravital images of a comparable ischemia-reperfusion–induced direct lung injury model, lung tissue with a warm ischemia duration of 45 minutes at 28°C was used and transplanted into syngeneic LysM-GFP mice. Intravital imaging was performed at the same imaging facility 2 hours after transplantation, with GFP positivity almost exclusively detected in monocytes (19).The acquired videos were analyzed using Imaris software, version 9.8.2 (Oxford Instruments).

### Histology and immunofluorescence staining.

For histological analysis, lung samples were placed inside a 20 mL syringe and inflated by creating negative pressure by pulling the syringe plunger, which was repeated several times until the lobes sank in 4% paraformaldehyde. After inflation, the lobes were fixed in 4% paraformaldehyde overnight at 4°C and then processed and embedded in paraffin. Alveolar wall thickness was examined in 4 μm sections from paraffin-embedded lung samples using a standard H&E staining kit (MilliporeSigma, HT107/HT109) as previously reported (6, 69). In short, alveolar walls in randomly selected lung regions that met the grid set by ImageJ were manually measured for wall thickness, and the average of at least 100 measurements was recorded for each sample. Immunofluorescence staining was performed on 7 μm fresh-frozen sections embedded into OCT compound (Thermo Fisher Scientific, 4585). In select experiments, 25 μL of 1 μm fluorescence microbeads (Invitrogen, Thermo Fisher Scientific, F13080) was i.v. injected into mice 10 minutes prior to sacrifice to assess stagnation in the microcirculation in the lung. Six images per lung were collected for blinded quantification by experienced laboratory members. OCT tissue cryosections were washed with PBST (0.05% Tween 20 in PBS) for 5 minutes at room temperature and permeabilized with 0.1% Triton X-100 in PBS for 5 minutes, followed by PBST washes (3 washes for 5 minutes each). After a 30-minute incubation in blocking buffer (1% BSA and 10% normal goat serum in PBS), sections were incubated with primary Abs overnight at 4°C, followed by PBST washes (3 washes for 5 minutes each), a 1-hour incubation at room temperature with secondary Abs (where appropriate), 5 minutes of exposure to Hoechst dye (Thermo Fisher Scientific, 62249), followed by PBST washes (3 washes for 5 minutes each) and application of mounting solution (2 mg/mL para-phenylenediamine). The following primary Abs were used: anti–mouse Gr-1 (FITC*-*conjugated, eBioscience, 14-5931); rabbit anti–mouse CD68 (Abcam, ab125212); rat anti–mouse CD41a (Invitrogen, Thermo Fisher Scientific, 14-0411-82); and rabbit anti–mouse fibrinogen (Abcam, ab34269). The following secondary Abs were used: Alexa Fluor 488 donkey anti–rat IgG (Invitrogen, Thermo Fisher Scientific, A21208); Alexa Fluor 594 goat anti–rabbit IgG (Thermo Fisher Scientific, A11037); and Alexa Fluor 546 donkey anti–rabbit IgG (Invitrogen, Thermo Fisher Scientific, A10040). For quantitative analysis, at least 5 representative areas that were captured with a Nikon Eclipse E800 fluorescence microscope at ×200 magnification were selected. In select experiments, F-actin in neutrophils was stained by rhodamine-conjugated phalloidin (Invitrogen, Thermo Fisher Scientific, R415) for 30 minutes at room temperature following fixation and permeabilization using 0.2% Tween 20.

### Statistics.

All results are reported as the mean ± SD. Comparison of 2 groups was performed using an unpaired, 2-tailed Student’s *t* test. Comparison of 3 or more groups was performed by 1- or 2-way ANOVA, followed by appropriate multiple-comparison tests, as indicated in the figure legends. Statistical analyses were performed using GraphPad Prism 10.1.1 for MacOS (GraphPad Software). *P* values of less than 0.05 were considered significant.

### Study approval.

All animal experiments were approved by the IACUC of the Washington University School of Medicine (protocols 19-0044 and 22-0105).

### Data availability.

The mouse scRNA-Seq datasets used in this study have been uploaded and are available in the Gene Expression Omnibus (GEO) database (GEO GSE249928, AKI-lung and GSE249242, lung transplant). All data presented in the graphs are provided in the Supporting Data Values file in the supplemental material.

## Author contributions

AH conceived and coordinated the study, and designed experiments with YK. YK designed, performed, and analyzed most of the experiments. LN, CL, and AS performed some of the animal experiments, tissue sectioning, and staining. YK, WL, and MJM performed the intravital imaging. SK performed arterial blood collection from mice under ventilation. HMS and DK provided the scRNA-Seq dataset of murine lung transplantation. AH and YK interpreted all the data, prepared the figures, and wrote the manuscript. EK, MJM, and DK critically revised the manuscript. AH and MJM provided experimental resources. AH supervised the entire project. All authors read and approved the final version of the manuscript.

## Supplementary Material

Supplemental data

Supplemental video 1

Supplemental video 2

Supplemental video 3

Supplemental video 4

Supporting data values

## Version 1 03/06/2025

In-Press Preview

## Version 2 05/15/2025

Electronic publication
